# Paternal well-being perception during childbirth: Experience of prepared Chilean fathers after a prenatal education intervention

**DOI:** 10.1590/1980-220X-REEUSP-2024-0009en

**Published:** 2024-10-28

**Authors:** Claudia Uribe-Torres, Mónica Muñoz Serrano, Paulina Bravo Valenzuela, Margarita Bernales Silva, Jade Bilardi, Meredith Temple-Smith

**Affiliations:** 1 Pontificia Universidad Católica de Chile, Facultad de Medicina, Departamento de Salud de la Mujer, Santiago, Chile.; 2 Fundación Arturo López Pérez, Santiago, Chile.; 3 University of Melbourne, Department of General Practice, Melbourne, Australia.

**Keywords:** Psychological Well-Being, Parturition, Father-Child Relations, Prenatal Education, Bem-Estar Psicológico, Parto, Relações Pai-Filho, Educação Pré-Natal, Bienestar Psicológico, Parto, Relaciones Padre-Hijo, Educación Prenatal

## Abstract

**Objective:**

To understand the father’s lived experience of childbirth as a significant life situation within the well-being concept framework. To understand the father’s lived experience of childbirth within the framework of the concept of well-being as a significant life situation.

**Method:**

Secondary data analysis from a qualitative study about the experience of twelve Chilean fathers who were prepared to actively participate at childbirth from a mixed public-private health system institution between 2016-2017, was carried out. Qualitative data were extracted from transcripts of open interviews with eight of the twelve fathers after childbirth. Data were analyzed using an interpretive-phenomenological approach.

**Results:**

Four central themes emerged from data, which were framed and understand within the psychological well-being concept: I. Feeling as a part of the healthcare team; II. Perceiving himself capable of containing and supporting his partner and being a guardian of the process; III. Being committed to being a father from the first moment of contact with the child; IV. Being wrapped in a whirlwind of emotions.

**Conclusion:**

Father’s lived experience at childbirth can be understood considering the psychological well-being concept. Prepared fathers could live the childbirth experience within a state of well-being, focusing on their achievements, commitments, and being satisfied with their roles as father and partner.

## RESUMO

**Objetivo:**

Compreender a experiência vivida pelo pai no parto sob o conceito de bem-estar como uma situação significativa da vida.

**Método:**

Foi realizada uma análise de dados secundários a partir de um estudo qualitativo sobre a experiência vivida do parto de doze pais chilenos, previamente preparados para participar ativamente no parto, entre 2016 e 2017 em uma instituição de saúde mista público-privada. Os dados qualitativos foram obtidos a partir da transcrição de entrevistas abertas no pós-parto com oito dos doze participantes e foram analisados usando uma abordagem fenomenológica-interpretativa.

**Resultados:**

Emergiram dos dados quatro temas centrais, enquadrados e compreendidos sob o conceito de bem-estar psicológico: I. Sentir-se parte da equipe de saúde; II. Perceber-se capaz de apoiar, dar suporte à sua parceira e ser um guardião do processo; III. Comprometer-se a ser pai desde o primeiro momento de contato com o bebê; IV. Estar envolvido em um turbilhão de emoções.

**Conclusão:**

A experiência vivida pelo pai no parto pode ser compreendida sob o conceito de bem-estar psicológico. Os pais com preparação prévia poderiam viver a experiência do parto em estado de bem-estar, centrados em suas conquistas, compromissos e satisfação com seu papel de pai e parceiro.

## RESUMEN

**Objetivo:**

Comprender la experiencia vivida del padre en el parto bajo el concepto de bienestar como situación significativa de la vida.

**Método:**

Se realizo un análisis de datos secundarios a partir de un estudio cualitativo sobre la experiencia vivida de parto de doce padres chilenos, previamente preparados para participar activamente en el parto, durante 2016 y 2017 en una institución de salud mixta público-privada. La información cualitativa se obtuvo de la transcripción de entrevistas abiertas en el posparto con ocho de los doce participantes. Los datos se analizaron utilizando un enfoque fenomenológico-interpretativo.

**Resultados:**

De los datos emergieron cuatro temas centrales, enmarcados y comprendidos bajo el concepto de bienestar psicológico: I. Sentirse parte del equipo de salud; II. Percibirse capaz de contener, apoyar a su pareja y ser un guardián del proceso; III. Comprometerse con ser padre desde el primer momento de contacto con el bebé; IV. Estar envuelto en un torbellino de emociones.

**Conclusión:**

La experiencia vivida por el padre en el parto puede ser comprendida bajo el concepto de bienestar psicológico. Los padres con preparación previa podrían vivir la experiencia del parto en estado de bienestar, centrados en sus logros, compromisos y satisfacción con su rol de padre y de pareja.

## INTRODUCTION

The birth process, since its origins, has corresponded to a social and family event, in which the arrival of a child consolidates the family figure for many couples^(1)^. It is common for men to participate in one way or another during the pregnancy and birth of their own children, which has become a key aspect to their benefit as individuals and as fathers^(2)^. Likewise, their participation benefits life and family project of the couple^(3)^.

The arrival of a child, and the process of establishing fatherly relationships require not only the physical presence of the father, but his social and emotional presence during those special moments of life, which could be transcendental to the relationship with his child^(4)^. A father who is present and active at birth is involved from the gestational stage, both in his role as a companion^(5)^ as well as his role as a father^(6)^. For other cultures, however, it would be enough to just be present fulfilling their social and safety responsibilities for their partner, without participating or being directly involved during labor or birth^(7, 8, 9)^ Whichever the case, fathers, and especially first-time fathers, require knowledge about pregnancy, birth, and parenting, to meet their expectation of supporting or relating to the child. Unfortunately, not all fathers are involved or prepared to play their roles during the prenatal stage, and even less so during the time of birth^(10,11)^. A father who does not have the adequate preparation for their expectations and needs could live this experience in a scenario full of negative emotions of fear, stress, and anxiety^(3,12,13)^, instead of living it as a fulfilling experience of well-being.

*Fathers’ involvement and well-being concept.* If the challenge is to include fathers in the role of co-parenting from early stages of pregnancy, and especially by being involved at birth, it is important to highlight fathers’ positive experiences as much as it has been done for mothers^(14)^ and, in the same way trying to understand the lived experience and the father’s well-being state. This concept has been widely defined and analyzed from historical philosophical perspectives and developed as a line of research within psychology^(15,16)^, however, it has not been possible to fully agree upon its definition or the nature of its structure^(16,17)^. Moreover, studies on the well-being experienced in significant life situations, such as the experience of fatherhood^(18)^, where health processes are intertwined, have been scarce.

Most authors have explained the concept of well-being, and its relationship to individual or social judgments, based on the constructs of life satisfaction and happiness^(15,17)^ Similarly, given that well-being has been linked to happiness and life satisfaction, authors have attempted to define well-being through a hedonic approach, focusing mainly on affectivity or positive emotionality, enjoyment or pleasure^(15)^. Accordingly, it has been described through an eudaemonic perspective, which, in addition to affectivity, associates satisfaction with personal development and growth to effort, meaning, achievements, and commitment, among others^(16)^

What has been reported thus far in the literature, and what could relate the concept of well-being to fathers’ experiences at birth, correspond to some of the elements that shape ­well-being, which have been explored independently. The case of paternal affectivity at birth has been studied and understood through qualitative methods^(19,20)^, while other elements that could be related to well-being have been approached from a quantitative perspective and considering the construct of “client satisfaction”^(12,13)^. On the other hand, the focus is, most of the time, on the experience of first-time fathers^(11,21)^.

Regardless of what the approach is, or from what approach the concept of paternal well-being is defined, what is proposed in this study is to understand, from a qualitative perspective, how fathers lived the experience of childbirth, and how they were involved in this process, within the framework of the concept of perceived well-being.

## METHOD

### Primary Study (PS) Design

Two studies with a qualitative approach were conducted between the Pontificia Universidad Católica de Chile and the University of Melbourne, Australia. Both studies aimed to explore the experience of fathers’ participation in labor and birth, asynchronously, in 2016 and 2020, respectively. This article reports on Chilean fathers’ experiences during the childbirth between 2016 and 2017^(6)^. A secondary supplementary data analysis^(22)^ of the primary study (PS) was conducted, to reveal the phenomenon of paternal experience at childbirth under another conceptual framework, different from that of the PS.

### PS Site

The study was carried out in a mixed public and private health system of a University Health Network in Santiago de Chile and was continued in the participants’ homes at the time of postpartum.

### PS Selection Criteria

Adult men, partners of pregnant women, were invited to participate in a prenatal education intervention (PEI) for fathers through Action Research. Information posters about the study and the contact of the researchers were placed in the health centers. Fathers were invited to live a paternal experience of labor and birth in their different roles after the PEI. Fathers who would not participate at childbirth were excluded.

### PS Sample Definition

It was a convenience sample from a total of 12 adult fathers, who were trained in the PEI. The intervention included focus groups to understand education needs of expectant fathers. After that, they participated in three to four educational sessions that focused on male parents’participation during pregnancy, childbirth, and postpartum period.

Four themes synthesized the PEI of the PS: fathers’ intention to establish contact with their baby from prenatal period; fathers’ needs to know more about childbirth; fathers’ desire to make physical father-child contact at childbirth; training how to care the baby during the first days after birth. After the prenatal intervention all of them were invited to share their lived experience of childbirth starting 2 weeks postpartum. The final sample for interviews was 8 fathers, since due to time issues 4 of the 12 could not participate. Despite this, the saturation criterion for this new approach to the phenomenon was met^(23)^

### PS Data Collection

Data was collected through 8 open in-depth interviews in the way face to face, which lasted an average of 50 minutes. All interviews were carried out by the principal researcher, who was a female PhD. The guiding open-ended question for fathers was: *Could you please share… How was the experience of participating* and *being involved in the birth of your child?*

All individual interviews prior participants authorization were recorded in an MP3 audio system and finally transcribed verbatim by a trained research assistance. Participants’ identities were kept confidential, and pseudonyms were used in the transcriptions of the interviews. Field notes were made by the researcher after each interview.

### Data Analysis and Treatment

Secondary Analysis was carried out by a Chilean-Australian researchers’ team (two Chilean coder and two Australian re-coder). The analysis procedure was also led by the principal investigator of the original study and no software was used for data processing. It was based on an interpretive paradigm and a phenomenological approach^(24)^; that allowed to understand the experiences lived by fathers under a new framework, based on the concept of well-being, since the phenomenon was observed as it was experienced and perceived by each participant. On the other hand, Husserl’s phenomenological approach allowed the discovery of “essential units” in the data^(25)^ that had not been revealed in the PS. In this way, through a phenomenological approach, the central categories emerged from this current secondary analysis of data could support the concept of well-being perceived by a father when they narrate their childbirth experience. To confirm member checking process main results summary was sent to the participants.

### Ethical Aspects

The research procedures were developed in accordance with international ethical regulations, and according to the legislation that regulates health research in Chile. Both the PS and the current secondary analysis were approved by the institutional Scientific Ethics Committee at the Pontificia Universidad Católica de Chile, (ID: 15-159; ID: 190313012) which validated the process and the Consent Informed Format of the PS. To protect the identity of the participants in the transcriptions, fancy pseudonyms chosen by themselves were used, while for the analysis data participants (P) were presented through alphanumeric coding, according to the chronological order of participation (P1, P2, P3, and so on).

## RESULTS

The reports of the original study came from eight male fathers with the following characteristics: Average age of 37, with ages ranging from 27 to 54. Of the total, six participants were first-time fathers. Regarding nationality, one was Argentine and the other seven were Chilean. Regarding their educational levels, four participants had professional technical degrees, two had complete high school education, and two had completed university higher education. Their socio-economic levels were determined by their health insurance system, where three participants had private health insurance, and five were dependent on public healthcare. Regarding their marital status, four of them were married and the other four were single cohabitants.

The information that emerged from the original interviews was analyzed and interpreted within the framework of the essential units that could characterize the fathers’ experiences at birth, and potential relationships between their experiences and perceptions of well-being.

It was observed that most fathers began narrating in a descriptive way and pointing out sequences of events. Later, as the interviews progressed, the fathers were transported to the moment of birth and relived the experience in a deeper way. From the phenomenological interpretative analysis of the narratives, each of the meanings (essential units) of paternal lived experiences, and their relationships with well-being were revealed.

**Figure 1:**
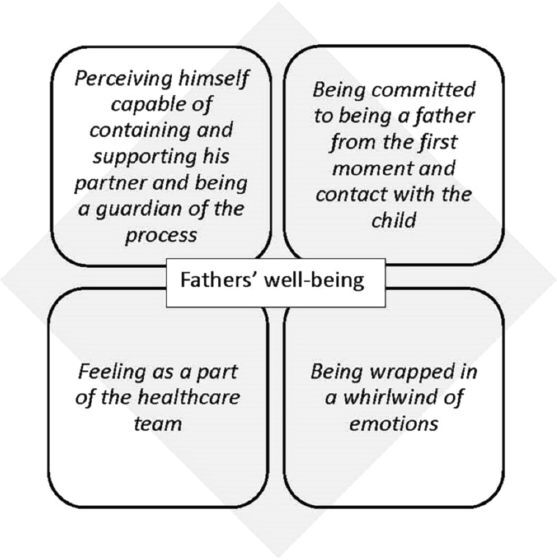
Core themes that could explain the parental well-being experience at childbirth.

The four central themes or categories that emerged in relation to the Chilean fathers’ lived experiences and well-being perception at childbirth are shown in Figure 1.

*I. Feeling as a part of the healthcare team*

The fathers’ narratives reflected a sense of feeling part of the healthcare team from two perspectives. The first indicates a form of satisfaction with respect to the reception of the health team, as in for example: “*So, she was very generous in the space (the midwife),* with the *sense of calm with which we lived it. We were quite lucky in that regard, because there was a lot of good disposition from everyone, so it was very good, it was very comforting”. (P7)*

It is also worth highlighting an aspect of empowerment from the fathers’ narratives, where they felt like active protagonists in the process, and felt the impulse to participate actively.

**Feeling included and integrated into the health team.** For some fathers, being able to put into practice what they learned, and being able to understand each of the procedures that were performed at birth was of great personal satisfaction.

*[…] I did not leave at any time because I went there (to the neonatology unit), they cleaned her, they did the tests they had to do, and I was there, and it was great, I felt part of everything. (P3)*

*A great satisfaction, I feel that I participated within what one can participate, and in what we had learned with the confidence that the workshops gave me. (P2)*

**Feeling that participation is lived in complicity with the health team.** For some fathers, a feeling of complicity with the healthcare team emerged, as if the fathers had felt that the professional team knew, valued, and became part of their competences and personal interests.

*There was a complicity (with the healthcare team), a planning of what we were going to do.—She is going to be born, we’re going to give her to the mother, then we’re going to examine her and we’re going to hand her to you, so calm down—(P1)*

**Feeling like obeying or receiving directions.** For some of them, however, receiving instructions from the healthcare team, prevented them from freely take on their roles, and made them an obedient participant with some degree of insecurity and nervousness. This could have made some fathers feel undervalued.

*They left me waiting outside […]I was a little nervous. Then they told me –put on these clothes– and there they told me – quickly! that we are already on the hour– (P4)*

*II. Perceiving himself capable of containing and supporting his partner and being a guardian of the process*

Much of the contents of the fathers’ narratives focused on the experience of providing support to their partners, placing their partners´ needs during labor at the center of attention. In such cases, they felt a responsibility and commitment toward their role in monitoring the labor process and the safety of their partner.

**Feeling helpful and able to guard and support his partner in labor.** For some interviewees, perceiving themselves as being useful, and participating in assisting and supporting the couple made them feel that their presence was relevant, both to accompany and to ensure the safety of their child and partner. This was described by fathers as a positive, special, and “magical” experience that left them very satisfied.

*And I felt useful, we knew what I had to do… I worried about the saline solution, and the anesthesia so they wouldn’t run out. So, I felt like I was participating, and of the birth itself too, for my part I felt part of it. I don’t know, it was special, everything was magical. […] I felt that she (the couple) trusted me. (P8)*

**Feeling worried and caring about the partner’s pain.** Some fathers revealed that the issue of pain was a concern, but it also encouraged them or mobilized them to try to make their partner in labor feel better by first trying by themselves or asking someone for help.

*I tried to help and calm her down a little bit, we had moments when the anesthesia… I mean, I was trying to help in any way possible and notifying the team of anything. (P2)*

**Feeling helpless from not being able to provide support or containment**. It was observed that the same situation that prompted some fathers to occupy themselves and to act, generated frustration in others, who felt that they were unable to do more to ease the pain of their partner, which became part of their own pain.

*I saw she was in a lot of pain and gave me… I do not know… sorrow. Obviously, it was for something, for something nice but just like seeing her that she was suffering and not being able to do anything but hold her, nothing could be done. (P1)*

*III. Being committed to being a father from the first moment of contact with the child*

It can be seen in Table 1 when fathers were able to have physical contact with their newborn child, according to the type of delivery.

**Table 1:** Types of the delivery and moment at which father-child contact was made – Santiago, Chile, 2016.

Participant	Type of delivery	Time of contact
*P1	Normal childbirth	At birth, prior to the immediate care procedures provided by the RN
*P2	Caesarean section due to maternal causes	During the first 6 hours
*P3	Cesarean section due to fetal causes	During the first 6 hours
*P4	Forceps due to fetal cause	During the first 6 hours
*P5	Caesarean section due to maternal causes	At birth, prior to the immediate care procedures provided by the RN
*P6	Normal childbirth	At birth, prior to the immediate care procedures provided by the RN
*P7	Normal childbirth	At birth, prior to the immediate care procedures provided by the RN
*P8	Forceps due to fetal cause	During the first 6 hours

Fathers expressed how their feelings were evolving from the moment of birth. The first sensations and emotions were generated at the time of the child’s first appearance. Anxiety and stress prevailed at the very moment of birth, when there was no certainty that the birth would be successful. However, after the birth occurred, for these trained participants, impulses toward fathering emerged when they held the child in their arms, and establish skin-to-skin, father-child contact, after the period of mother-child contact. In that moment previous worried emotions turned to feelings of tenderness and peace.

**Feeling that they can carry the child naturally and calmly**. Some participants felt compelled to hug and hold their child with intimate contact, as if an internal, innate force was guiding them. Touching and being able to talk to their babies became a natural and spontaneous tendency. They felt that they were providing calm and tranquility.

*I didn’t feel afraid to take her! It was… immediately everything was natural. […] the most beautiful thing was that she was born, well as her crying. When I held my daughter in my arms, she was quiet, she was super calm, relaxed. (P1)*

**Perceiving that through physical contact (bonding) they could transmit care and protection to their child.** For many fathers, physically contacting their child represented a very special moment for them, and they perceived that physical contact generated wellbeing to their babies, which was a comfortable feeling of tranquility and security. Some felt that they were able to pass love and protection through skin-to-skin contact.

*Once I had her on my chest skin to skin, she stood calm…in a nutshell it is like feeling protected and loved. (P3)*

*I realized that she was so helpless […] she was very close to me, as if she asked me for affection and protection. (P6)*

**Feeling a direct connection and father-child synchrony through physical contact**. For fathers, physical contact with the child was a way to establish a deep connection that transcended the merely physical realm. From the moment they saw their child for the first time, an initial connection was established, but touching them established a deeper connection, and there was even a synchronization of breathing between the father and baby. The sensory stimuli that were generated through skin contact allowed them to connect recognize each other.

*From the minute I saw my baby, I don’t think he looked at me but if I felt a connection, I think it was automatic for me to take him, the touch gives you… an emotional connection, a connection of feelings more than something physical. (P2)*

*When bonding was happening, I was feeling him on my chest and he felt calm. It was an exciting union; it felt as if our breath was one. My breathing was not normal, it was like accelerated, I was completed connected with him, it was fantastic. (P8)*

**Perceiving that the child recognizes him as a “dad”.** For some fathers, the experience of feeling recognized by their child was very relevant. The moment of direct physical contact between them corresponded to the instance in which they tested if they could be recognized by contact with the hands or by voice.

*I took him in my hands and it was a sacred moment, he would recognize my hands, my voice […] with his eyes open, as if to say –here I am, I came into the world– I felt that, and I was very struck by that, as if he had said –this is my dad–. (P5)*

**Feeling the child’s life through contact.** A very particular sensation transmitted in some of the narratives corresponded to the satisfaction of perceiving the life of the child, through the breath, the heartbeat, and the temperature of the skin. Fathers felt great about being able to perceive chest movements (breathing movements), which accounted for their child’s vitality.

*I don’t know how to describe it. I felt his warmth and he was moving a little; I felt his heart, that is, it was more the movement, the movement of the little chest with the breath. (P2)*

*I was happy that she felt comfortable and safer; she felt the protection that was there - that more than anything, to feel her breathe too, it gave me joy that she was breathing. (P4)*

**Feeling how the commitment to be a father was sparked from birth**. The moment of birth in general, and especially the moment of the father-child encounter, was transformed into a pact for the fathers, as well as a commitment to fathering and responsibility in care.

*I’m going to be forever grateful that I was able to spend those 15 to 20 minutes with her in proximity. Contact is something that doesn’t have a price. I guess that will grow and will be strengthened in the future. (P7)*

*She was there [the baby], and I loved her very much. That made me feel ready, like now I must take care of her forever; like that’s why I didn’t pay attention to other details. (P1)*

*IV. Being wrapped in a whirlwind of emotions*

The fathers revealed in their testimonies that, regardless of how useful the prenatal preparation was, they did not feel empowered enough to manage and control their emotions. The event of childbirth, as a borderline experience for the mother, was more so for the father. They felt that, despite having enough information about the birth process, they did not have control of it. This whirlwind of sensations and emotions, although they were mostly positive, reflected the state of affective well-being of the fathers. States of tranquility and pleasure turned to states of greater nervousness and stress, and vice versa, as the moment of birth approached.

**Experiencing the uncertainty of not knowing how to act.** The experience reported by a father who failed to position himself in his role as a companion during the first stage of labor revealed the nervousness experienced, not knowing how to act when he only received indications from healthcare team, and they were contradictory.

*A little nervous, just because there were many people, and everyone told me –stay there! – it was like… I didn’t know where to position myself because one person told me something, and another told me something different. (P4)*

**The experience of contradicting emotions when facing birth and fatherhood.** During the moment of birth, positive, and negative emotions and sensations were experienced simultaneously. Emotions such as fear, anxiety and uncertainty were related to the outcome of the birth, and the health of the newborn. The crying that represented the vitality of the newborn was a fundamental element that allowed fathers to turn the state of anxiety and uncertainty into a state of tranquility and well-being. In the latter state, they were able to enjoy the moment, reliving the moment as being magical and indescribable.

*My life changed, to look at her, to observe her movements, her crying… I was in the clouds. It is indescribable the happiness that it made me feel. I felt very serene. (P3)*

**Focusing his attention only on his child – a magical moment that disconnects him from the environment**. In their narratives, the fathers referred to a magical and peaceful experience that is difficult to reproduce. The encounter with their newborn made them distance themselves and disconnect from the entire environment, to focus their attention only on the emotion generated by the presence of their baby.

*I loved seeing my daughter come out, I went inward […] I tended to disconnect [from the environment]. I focused so much on the moment that I… I was filled with so many emotion… a lot. It was a unique sensation -an experience that can´t be transmitted. But the truth is that they cannot be reproduced…all emotions at that time. It was a very magical moment. (P6)*

*It was an incredible sensation of peace. There was nothing else around. It was a very emotional moment, actually!! (P5)*

## DISCUSSION

This study was based on the narratives of men who lived a birth experience, for which they had prepared during the pregnancy stage. This prenatal intervention focused on fathers’ own interests and expectations. Hence, the findings of this study could be related to fathers who are involved from pregnancy, rather than the more “common fathers”, who generally feels invisible, without any control over the birth process^(26)^, and with a secondary role in this life event^(7)^.

A relevant aspect of this study was that it unearthed experiences lived by Chilean fathers from the perspective of subjective well-being. This can be deemed relevant, given that much of the available literature has described fathers’ experiences of birth through the lens of satisfaction felt by the fathers as “clients”. The latter is closer to the judgment or opinion that the father makes about the external conditions that come from the environment during the birth experience^(11)^. Thus, it is a great challenge to discuss the results of this study based on the revelation of a phenomenon seen from a different way than what has typically been described in the literature.

Four central categories emerged from Chilean fathers’ narratives of their experiences of well-being during their child’s birth. Such categories were reflected by judgments that each father made about his personal performance, as well as their sensations, emotions, and involvement in the process. Hence, fathers were able to explain the paternal experience at birth as a significant life situation, framed around the concept of well-being^(18)^ which is more from an eudaemonic perspective related to achievements, commitment, and involvement, than from a hedonic stance (sensations/emotions related to enjoyment and pleasure)^(15,16)^.

One of the central themes or categories, namely *Feeling as a part of the healthcare team*, could be understood both from the perspective of well-being, and the concept of user satisfaction. On the one hand, under the concept of user satisfaction, fathers described how they perceived themselves participating in the system as collaborators during the labor process. From the perspective of well-being, they felt capable and empowered with the health team. Fathers, as other authors point out, could feel that they participated in the event, when the health care provider integrated^(27)^ and welcomed them^(9,28,29)^. Perhaps the difference that is reflected in this Chilean study, compared to what other authors have described, could be related to fathers’ motivation and empowerment, which resulted from the preparation and special intervention they received before birth.

The theme or category of *Perceiving himself as being capable of containing and supporting his partner and being a guardian of the process* could also be understood from the eudaemonic well-being perspective, as it relates to the role of a companion. The participant of this study, as reported above, felt that they –by themselves– were useful in providing support to their partner, which to them was fundamental. This agrees with what other authors have pointed out regarding the role of guardian, caregiver and support of the couple during childbirth^(9)^. From the perspective of user satisfaction, it has been reported that when fathers felt they had made a positive difference in the birth and delivery process of their partners, they expressed greater satisfaction^(11)^. Additionally, other fathers reported dissatisfaction when they had to work hard to feel included and involved in supporting and containing their partner^(27)^. There have also been reports of fathers feeling excluded from the process altogether^(30)^.

Fathers in this study felt that they were able to collaborate in overseeing and ensuring the safety of their partner and their child during birth. This contributed directly to the understanding of the phenomenon of paternal well-being, while in other studies the level of fathers’ satisfaction related to the safety and health of the partner and newborn has focused exclusively on the action and competencies of the health team^(28,29)^. Regardless of whether interpretations are from the lens of well-being or user satisfaction, studies agree that fathers who do not perceive that everything is under control^(26)^, especially when complications occur during the process, generate feelings of frustration and incompetence in providing support to the partner^(19)^.

One of the issues that deserves to be highlighted in this discussion concerns paternal well-being from the lens of eudaemony, specifically regarding the role of the father. The latter corresponds to the category of *Being committed to being a father from the first moment of contact with the child*. The core of this category was that fathers felt they could connect with their child through contact and commit to caring for, holding, and calming them, more than just feeling a physical connection. There is currently little evidence of well-being or lived experience focusing on the meaning of fatherly at the time of physical father-child contact^(31)^. The only studies reporting an active father role during the moment of contact with his child were in the situation that mothers being disabled during childbirth^(32,33)^. These studies mainly described benefits for the newborn, rather than for men and fathering related on emotions and feelings^(31)^.

The fourth theme, namely *Being wrapped in a whirlwind of emotions*, has been most frequently reported by other authors as experiencing a dynamic scenario of multiple emotions, typically described as a “carousel of emotions”^(26)^. For most studies, the emotions experienced by fathers who have not received adequate preparation have been predominantly negative, and related to fear, stress, anguish, and frustration^(12,20,28)^. In this Chilean study, nevertheless, the “whirlwind of emotions”, which also emerged in their narratives, tended to focus more on the positive results of emotional well-being, referred to as peace, serenity, and happiness, rather than on the negative emotions of fear and anxiety.

This study is a contribution to the emergence and promotion of positive and present fathering. It also contributes to unveiling new male experiences, and different roles within the family group.

Two major limitations of this study need to be addressed. The first corresponds to the fact that some participants were first time fathers and others had previous experience. Hence, the data reported by the Chilean fathers could present biases, given that the information was analyzed as a whole group, without distinguishing participants with previous birth experiences.

The second aspect to consider as a limitation is that only trained fathers participated in this study. Thus, participants in this study do not correspond to the general population of Chilean partners who typically live a birth experience without preparation.

As a strength of this study, it could be noted that this is the first exploratory effort in the Latin American region to gather fathers’ experiences from the lens of well-being, rather than that of user satisfaction. From this starting point, it could be possible for nursing field to design an instrument in the future to collect fathers’ perceptions of well-being during childbirth, where the main dimensions derive from the four central themes or categories revealed in this study.

## CONCLUSION

Father’s lived experience at Childbirth could be explained considering psychological well-being concept. Emotions and sensations typical of hedonic well-being were perceived by the trained parents around the moment of birth, while everything related to the eudemonistic approach, such as commitment, achievements, and satisfaction with respect to parental roles, were revealed once the birth had occurred.

## References

[1] García-Portuguez VA, Muñoz-Serrano M, Uribe-Torres C (2020). Father committed to early parentem from the first father-child contact experienced at birth. Aquichan.

[2] Maldonado-Durán M, Lecannelier F (2008). El padre en la etapa perinatal. Perinatol Reprod Hum.

[3] Vischer LC, Heun X, Steetskamp J, Hasenburg A, Skala C (2020). Birth experience from the perspective of the fathers. Arch Gynecol Obstet.

[4] Krampe E, Newton R (2006). The father presence questionnaire: a new measure of the subjective experience of being fathered. Fathering.

[5] Muñoz-Serrano M, Uribe-Torres C, Hoga L (2018). Fathers prepared for and committed to their role as companions during the birth process. Aquichan.

[6] Uribe-Torres C, Muñoz M, Hoga L (2021). Father prepared, committed, and involved in his child’s birth: the experience of early father-child skin to skin contact at birth. Open J Obstet Gynecol.

[7] Abushaikha L, Massah R (2012). The roles of the father during childbirth: the lived experiences of arab syrian parents. Health Care Women Int.

[8] McLean KE (2020). Men’s experiences of pregnancy and childbirth in Sierra Leone: reexamining definitions of “male partner involvement”. Soc Sci Med.

[9] Longworth MK, Furber C, Kirk S (2021). Fathers’ roles matter too: an ethnographic study examining fathers’ roles and the influences on their roles during labour and birth. Midwifery.

[10] Xue WL, He HG, Chua YJ, Wang W, Shorey S (2018). Factors influencing first-time fathers’ involvement in their wives’ pregnancy and childbirth: a correlational study. Midwifery.

[11] Howarth AM, Scott KM, Swain NR (2019). First-time fathers’ perception of their childbirth experiences. J Health Psychol.

[12] Hildingsson I, Haines H, Johansson M, Rubertsson C, Fenwick J (2014). Childbirth fear in Swedish fathers is associated with parental stress as well as poor physical and mental health. Midwifery.

[13] Saxbe D, Horton KT, Tsai AB (2018). The birth experiences questionnaire: a brief measure assessing psychosocial dimensions of childbirth. J Fam Psychol.

[14] Uribe C, Contreras A, Villarroel L, Rivera S, Bravo P, Cornejo M (2008). Bienestar materno durnate el proceso de parto: desarrollo y aplicación de un escala de medición. Rev Chil Obstet Ginecol.

[15] Diener E, Lucas RE, Oishi S, Snyder CR, Lopez SJ (2002). Handbook of positive psychology [Internet].

[16] Huta V, Waterman AS (2014). Eudaimonia and its distinction from hedonia: developing a classification and terminology for understanding conceptual and operational definitions. J Happiness Stud.

[17] Gasper D (2010). Understanding the diversity of conceptions of well-being and quality of life. J Socio-Economics.

[18] Lauinger W (2015). A framework for understanding parental well-being. Philosophia.

[19] Hasman K, Kjaergaard H, Esbensen BA (2014). Fathers’ experience of childbirth when non-progressive labour occurs and augmentation is established. A qualitative study. Sex Reprod Healthc.

[20] Hanson S, Hunter LP, Bormann JR, Sobo EJ (2009). Paternal fears of childbirth: a literature review. J Perinat Educ.

[21] Ledenfors A, Berterö C (2016). First-time fathers’ experiences of normal childbirth. Midwifery.

[22] Heaton J (2008). Secondary analysis of qualitative data: an overview. Hist Soc Res (Koln).

[23] Guest G, Namey E, Chen M (2020). A simple method to assess and report thematic saturation in qualitative research. PLoS One.

[24] Duque H, Aristizábal Díaz-Granados ET (2019). Análisis fenomenológico interpretativo. Pensando Psicología.

[25] Husserl E, Husserl E (2016). La Idea de la Fenomenología.

[26] Etheridge J, Slade P (2017). “Nothing’s actually happened to me.”: the experiences of fathers who found childbirth traumatic. BMC Pregnancy Childbirth.

[27] McNab E, Hollins Martin CJ, Norris G (2022). Factors that influence father’s experiences of childbirth and their implications upon postnatal mental health: a narrative systematic review. Nurse Educ Pract.

[28] Johansson M, Rubertsson C, Rådestad I, Hildingsson I (2012). Childbirth – An emotionally demanding experience for fathers. Sex Reprod Healthc.

[29] Premberg A, Carlsson G, Hellström AL, Berg M (2011). First-time fathers ’ experiences of childbirth. A phenomenological study. Midwifery.

[30] Steen M, Downe S, Bamford N, Edozien L (2012). Not-patient and not-visitor: a metasynthesis fathers’ encounters with pregnancy, birth and maternity care. Midwifery.

[31] Cordolcini L, Castagna A, Mascheroni E, Montirosso R (2024). Skin-to-skin care and spontaneous touch by fathers in full-term infants: a systematic review. Behav Sci (Basel).

[32] Erlandsson K, Christensson K, Fagerberg I (2008). Fathers ’ lived experiences of getting to know their baby while acting as primary caregivers immediately following birth. J Perinat Educ.

[33] Velandia M, Uvnäs-Moberg K, Nissen E (2012). Sex differences in newborn interaction with mother or father during skin-to-skin contact after Caesarean section. Acta Paediatr.

